# Development of a ZRS Reporter System for the Newt (*Cynops pyrrhogaster*) During Terrestrial Limb Regeneration

**DOI:** 10.3390/biomedicines12112505

**Published:** 2024-11-01

**Authors:** Martin Miguel Casco-Robles, Ryosuke Ikeda, Fumiaki Maruo, Chikafumi Chiba

**Affiliations:** 1 Faculty of Life and Environmental Sciences, University of Tsukuba, Tennodai 1-1-1, Tsukuba 305-8572, Ibaraki, Japan; maru@biol.tsukuba.ac.jp (F.M.); chichiba@biol.tsukuba.ac.jp (C.C.); 2 Graduate School of Science and Technology, University of Tsukuba, Tennodai 1-1-1, Tsukuba 305-8572, Ibaraki, Japan; s2220917@u.tsukuba.ac.jp

**Keywords:** newt, limb regeneration, Shh, Schwann cell, pattern formation, ZPA, ZRS

## Abstract

Background: Newts, a type of urodele amphibian, offer remarkable insights into regenerative medicine due to their extraordinary tissue regeneration capabilities—a challenging feat in humans. During limb regeneration of adult newts, fascinating cellular and molecular processes are revealed, including scarless healing, de-differentiation of mature cells, and regeneration of limbs and digits. Sonic hedgehog (Shh), crucial for vertebrate limb development, is regulated by the zone of polarizing activity regulatory sequence (ZRS) in the limb bud zone of polarizing activity (ZPA). The metamorphosed (terrestrial) newt can reactivate Shh during regeneration, facilitating proper limb patterning. Cell types capable of regulating the ZRS in metamorphosed newts remain unknown. The identification of such cell types provides invaluable insight into novel regenerative mechanisms. Objective: In this study, we developed the first newt ZRS reporter. Methods: We isolated and characterized the newt ZRS enhancer (nZRS), identifying conserved DNA binding sites. Several binding sites with medical relevance were conserved in the newt ZRS. In functional analysis, we developed a system composed of a transgenic nZRS reporter newt and a new newt anti-Shh antibody, which allowed Shh monitoring during limb regeneration. Results: We identified a group of Schwann cells capable of ZRS reporter and Shh protein expression during terrestrial limb regeneration. Conclusions: This system provides a valuable in vivo approach for future genetic studies of patterning during limb regeneration.

## 1. Introduction

Following trauma, the metamorphosed adult newt (a urodele amphibian) can repair tissue with scar-less regeneration in several body parts: the lymphatic system, heart, jaw, brain, limb, retina, and lens [1,2,3,4,5,6,7,8]. In contrast, humans undergo fibrosis following injury in multiple tissues, ultimately leading to a progressive loss of function [9,10]. Following limb amputation, the wound site is quickly sealed by the wound epidermis and forms a mass of mesenchymal cells called the “blastema” [8,11,12]. There are two cellular mechanisms for limb regeneration that contribute to the newt blastema: a larval mode composed of stem cells and an adult terrestrial mode (post-metamorphosis) involving mature cell dedifferentiation [2,8]. The blastema is highly innervated and irrigated with blood vessels that allow blastema growth [13,14]. Urodele limb regeneration is a “nerve-dependent” characteristic observed in other organisms (fish, echinoderms, and annelids) during regeneration [14].

The adult blastema regulates proper patterning of the regenerated limb and digits [11], regardless of the proximodistal amputation site on the limb, referred to as the positional memory/identity [11,12]. In adult newts, the Prod 1–nAG signaling system is involved in blastema proximodistal identity [14,15,16]. The newt anterior gradient protein (nAG) is expressed in Schwann cells (SCs) [14]. In the case of the anterior-posterior and dorsal-ventral axes, the mechanism of limb patterning in metamorphosed limb regeneration remains unclear. Previous studies have shown that the adult newt blastema expresses Sonic hedgehog (Shh) in a localized posterior region, similar to the chick and mouse zone of polarizing activity (ZPA) of the limb bud [17,18,19]. During embryo development, the establishment of the developing ZPA by mesoderm Shh cells provides signaling for the anterior-posterior axis and is regulated by the zone of polarizing activity regulatory sequence (ZRS) enhancer of Shh [17]. The ZRS enhancer sequence is *cis*-acting with long-range capacity, playing a major role in the morphological evolution of vertebrate limbs and digit formation [20,21]. Differences between the larva and post-metamorphosed newt are seen during digit patterning. During larval digit development and regeneration, the first digits to form are digits one and two near the anterior, known as the preaxial dominance, later following metamorphosis (beyond 1 year of age and approximately 5–6 cm in body length), regenerating digits that resemble the amniote autopodium are patterned [2,22]. Grafting the adult newt ZPA of the blastema in a reversed manner led to a double Shh signal that yielded supernumerary limbs [11,19], resembling the classic mirror image experiments of the chick wing [18]. To investigate the sources of this blastemal ZPA signaling, intact newt limb skin graft experiments were carried out, followed by limb amputations [23]. In some cases, these skin manipulations led to multi-digit or mirror digits, and the conclusion drawn was that subcutaneous tissue was involved during digit regeneration [23]. A fundamental question remains unanswered. What adult intact cell types in metamorphosed newts regulate the ZRS following limb amputation to form the ZPA? Comparing such cell types in mammalian systems provides hope for developing newt-like regenerative strategies. Adult cells capable of regulating the ZRS enhancer of Shh beyond limb development have the potential in regenerative medicine to assist in the repatterning of tissue along the anterior-posterior axis of an amputated limb. Therefore, insight into Shh regulation via the limb-specific ZRS enhancer is important. Currently, there are no ZRS reporter tracking systems for the regenerating newt, *Cynops pyrrhogaster*. Therefore, in this study, we focused on the development and description of a new newt ZRS (nZRS) reporter in combination with a newt anti-Shh antibody for studying Shh expression during terrestrial limb regeneration. In vivo visualization of the nZRS reporter in metamorphosed newts is a powerful resource for investigating the spatial and temporal regulation of Shh+ cells during limb regeneration, providing insight into the field of regenerative medicine. Following metamorphosis, we identified a group of Shh+ SCs and skin glands capable of driving the ZRS reporter and expressing the Shh protein.

## 2. Materials and Methods

All experiments were carried out in accordance with the University of Tsukuba’s Regulations on Animal Experimentation. All genetic modification experiments were approved by the Genetic Modification Safety Committee of the University of Tsukuba (approval number 170110). The methodology was carried out and reported according to the ARRIVE guidelines 2.0 [24].

### 2.1. Animals

Adult males and females of the Toride-Imori line of Japanese fire-bellied newts (*C. pyrrhogaster*) were used in this study [2,25]. In brief, newts were maintained at 18 °C inside plastic containers that contained clean, shallow water and exposed to natural lighting. Females and males were kept in separate containers until the start of transgenic experiments. Animals were fed frozen mosquito larvae (Akamushi; Kyorin Co., Ltd., Himeji, Japan) daily, and mating tanks were cleaned regularly. Fertilized eggs at the 1-cell stage (F0) were obtained using an established protocol [25], and stages of development were scored using an established table [26]. Wild-type (WT) embryos were set aside from the same clutch for whole-mount staining or immunohistochemistry (IHC).

### 2.2. Anesthesia and Surgical Dissections

An anesthetic, FA100 (4-allyl-2-methoxyphenol; DS Pharma Animal Health, Osaka, Japan), was dissolved in Holfreter’s solution (pH 7.2–7.6). To administer the anesthetic, animals were submerged in dilute concentrations and kept in small plastic containers. The following concentrations of FA100 were used (*v*/*v*): larval stages 38–59 (0.025%, in 0.1× Holfreter’s); metamorphosed-juvenile newts 12–13 months, 5.5–6 cm (0.05% in 0.6× Holtfreter’s), adults 9–10 cm (0.1% in 0.6× Holtfreter’s). Limbs were amputated at mid-stylopod or mid-zeugopod stages using a cryostat blade (C-35, Feather, Osaka, Japan). Any protruding bone following amputations was trimmed with conventional surgical scissors. During limb regeneration of metamorphosed newts, individuals were placed in separate containers containing moist paper towels and cleaned regularly. Adult newt eyeballs were collected using a previously established technique [1].

### 2.3. Molecular Cloning of the Newt nZRS Enhancer Region

Newt genomic DNA was extracted from adult tail tips using the Wizard Genomic DNA Purification Kit (Promega, Madison, WI, USA) and kept at 4 °C until use. To isolate the newt nZRS, degenerate primer sets were designed by aligning known ZRSs of the following species: *Cynops orientalis* accession number MW653930.1 and *Pleurodeles waltl* accession number LC378706.1. Forward primer (5′GGTTCTACCTTMATATGTCGATCTT3′) and reverse primer (5′CGTGAAAATARCTGTTGAAATATCAC3′) were used. Genomic PCR was carried out using the Tks Gflex ™ DNA Polymerase Kit (R060A, Takara, Kusatsu, Japan) according to the manufacturer’s instructions. An 819-bp PCR product was obtained, treated for TA ligation with the Mighty TA-cloning Reagent Set for PrimeSTAR (6019, Takara, Kusatsu, Japan), subcloned into a pCR2.1 TOPO TA cloning vector (Life Technologies, Carlsbad, CA, USA), transformed into Stbl3 cells (Invitrogen, Carlsbad, CA, USA), then cultured at 30 °C overnight. Plasmid DNA containing the newt nZRS 819-bp region was purified with the GeneJET Plasmid Miniprep Kit (GeneJET, Carlsbad, CA, USA). The plasmid was linearized with conventional restriction enzymes (*KpnI* or *SpeI*) and sequenced with MinION (MIN-101B, Flow Cell (R10.4.1) FLO-MIN114, SQK-LSK114 sequencing kit, MinKNOW Stand-alone NC Windows version 23.11.5; Oxford Nanopore Technologies plc., Oxford, UK). The nZRS sequence is available at Genbank with accession number PP691624.

### 2.4. Molecular Cloning of C. pyrrhogaster Shh and Antibody

A contig for the newt Shh was found in the *C. pyrrhogaster* comprehensive transcriptome database, TOTAL (http://antler.is.utsunomiya-u.ac.jp/imori/, accessed on 29 March 2022) [13] (Figure S1). The existence of this Shh transcript in the limb blastema was detected by standard PCR-based molecular cloning (Figure S1a) using cDNAs that were constructed from stage III forearm blastemas using the Nucleospin RNA Mini kit for RNA purification (Takara, Kusatsu, Japan) and the SuperScript IV First-Strand Synthesis System (Thermo Fisher Scientific, Vilnius, Lithuania). A single 1.7 kbp PCR product was detected and subcloned for sequencing using the method noted above. The newt Shh nucleotide sequence is available at Genbank with accession number PQ306330. Based on the newt Shh nucleotide sequence (Figure S1b), a rabbit polyclonal antibody against *C. pyrrhogaster* Shh was generated (Eurofins genomics, Tokyo, Japan). Note that the epitope site targets the Shh signaling domain (N-Shh) and is upstream of the conserved site for internal auto-proteolytic cleavage (Figure S1b). Anti-Shh specificity was confirmed by IHC of the newt retina (Figure S1c). It was previously shown that the fish retina expresses Shh [27]. Therefore, the retina was used as a positive reference for anti-Shh validation. The newt anti-Shh was validated and detected in regenerating limb tissue but not in intact limbs of the adult newt (Figure S2). 

### 2.5. Bioinformatic Analysis

Version 5.5.4 of MEME Suite (https://meme-suite.org/meme/tools/meme, accessed on 18 October 2023) [28] in classic mode was used to align vertebrate ZRS conserved block regions. The nZRS sequence was aligned to the ZRS sequence of the following vertebrate species (accession number): *Homo sapiens* (NG_042169.2); *Canis lupus* (CP050577.1); *Mus musculus* (NG_042170.2); *Aquila chrysaetos* (LR606183.1); *Gallus gallus* (JN051259.1); *Anolis carolinensis* (AnoCar2.0v2); *Kaloula pulchra* (KY158906.1); *Xenopus tropicalis* (NM_001011160.1); *Ambystoma mexicanum* (AmexG_v6, Amex_PQ.v4) [29,30]; *Amolops loloensis* (MW653922.1); *Liua shihi* (MW653935.1); *Pseudobranchus axanthus* (MW653939.1); *Amphiuma means* (MW653924.1); *Cynops orientalis* (MW653930.1); and *Pleurodeles waltl* (LC378706.1). Vertebrate ZRS sequences were screened for additional conserved binding sites using JASPAR (https://jaspar.elixir.no/, accessed on 30 October 2023) using a default threshold of 80% [31]. The complete list of conserved alignment regions (see Tables S1–S8) was summarized in Figure S3 using the mouse ZRS. The ZRS sequences of vertebrates listed above were aligned using Pro-coffee MSA (version 11.00; https://tcoffee.crg.eu/apps/tcoffee/do:procoffee, accessed on 6 July 2024) [32].

### 2.6. Construction of the nZRS Reporter

The cpRPE65 promoter was released from the plasmid cpRPE65-mcherry01 [33] using *AjuI*/*KpnI*. The nZRS (819 bp) and human B-globin minimal promoter (hmp, 51 bp) [21,34] were positioned upstream of the mCherry reporter with the In-fusion HD cloning Kit (639648, Takara, Kusatsu, Japan). The following primer sets (5′ to 3′) were used (the underlined region contains the human minimal promoter; lowercase nucleotides represent infusion homologous sites): nZRS-hmp fragment 1 (forward primer tatggcatatgttgcGGTTCTACCTTCATATGTCGATCTT and reverse primer agatggctctgccctgactTTTATGCCCAGCCCCGTGAAAATAGCTGTTGAAATATC); hmp-mCherry backbone fragment 2 (forward primer agtcagggcagagccatctATTGCTTACATTGCTTCTcgcgggcccgggatc and reverse primer gcaacatatgccataTGCTGGCTGCCATGAACAAAGGTGG). PCR was carried out using the Tks Gflex ™ DNA Polymerase Kit (R060A, Takara, Kusatsu, Japan). This plasmid (a gift from Gary Felsenfeld at the National Institutes of Health, Bethesda, MD, USA) contains two dual-core chicken HS4 insulators flanking the target transgene to reduce the positional genomic effect and two *I-SceI* sites for newt transgenesis. In brief, the newt nZRS reporter plasmid contained the following elements and orientation: p*I-SceI*-2XHS4-nZRS-hmp>mCherry-pA-2XHS4-*I-SceI*. In short, hereafter, it is referred to as nZRS-hmp-mcherry01.

### 2.7. Newt Transgenesis

Newt transgenesis was carried out using a newt transgenic protocol [25,26]. In brief, fertilized de-jellied embryos at stage 1 were co-injected with *I-SceI* and plasmid DNA (nZRS-hmp-mcherry01). The microinjection volume was fixed at 2 nL. The microinjection mix contained the following: *I-SceI* (catalog #R06945; New England Biolabs, Tokyo, Japan), 1 U μL^−1^; *I-SceI* buffer (New England Biolabs), 1×; plasmid DNA, 50 or 100 ng μL^−1^. Uninjected WT embryos were kept as controls and reared beyond metamorphosis along with nZRS reporter transgenic newts. Transgene insertion into the genome was confirmed using primers for the nZRS region (forward primer 5′GGTTCTACCTTCATATGTCGATCTT3′) and mCherry region (reverse primer 5′TTACTTGTACAGCTCGTCCATGCCG3′). Transgenic newts at larval stages 57–59 were screened by collecting genomic DNA from tail tips.

### 2.8. Tissue Fixation

The fixative 3% glyoxal (078-00905, Wako; Fujifilm, Osaka, Japan) [35,36] and 2% paraformaldehyde (PFA) in PBS (pH 7.0, adjusted with acetic acid) was stored in an amber-colored glass bottle at 4 °C and used within 1 month. All fixations were carried out at 4 °C. Newt embryos were fixed at limb bud stages 38–40 for 4 h, metamorphosed newt limbs for 6 h, adult eyeballs for 6 h, and adult limbs for 15 h. The fixed samples were washed several times for 3 h, each wash in PBS at 4 °C; limbs were decalcified in 10% ethylenediamine-N,N,N′,N′-tetraacetic acid (EDTA) in PBS (pH 7.0, adjusted with NaOH) for 48 h at 4 °C, rinsed in PBS, then transferred to 30% sucrose in PBS at 4 °C. Following equilibration in a 30% sucrose solution, samples were embedded into an O.C.T. compound (4583; Sakura Finetech, Tokyo, Japan) and sectioned to 20 µm thickness using a cryotome. Frozen sections were air dried in the dark for 24 h before direct observation of their fluorescence or immunohistochemistry.

### 2.9. Immunolabeling

For the immunohistochemistry of sections, the following primary antibodies were used: mouse anti-RFP monoclonal antibody (1:500; AKR-021; Cell Biolabs, San Diego, CA, USA), anti-acetylated tubulin monoclonal antibody (1:500; T6793; Sigma-Aldrich, Saint Louis, MO, USA), rabbit anti-Shh polyclonal antibody (1:500) (Figure S1), anti-NCAM polyclonal antibody (1:500; AB5032; Chemicon, Sigma-Aldrich, Darmstadt, Germany), and anti-RFP polyclonal antibody (1:500; 600-401-379; Rockland Immunochemicals, Limerick, PA, USA). Secondary antibodies were: Alexa 488-conjugated goat anti-mouse IgG (H + L) (1:500; A11001; Thermo Fisher Scientific, Carlsbad, CA, USA), rhodamine (TRITC)-conjugated affiniPure goat anti-mouse IgG (H + L) antibody (1:500; 115-025-062; Jackson ImmunoResearch, West Grove, PA, USA), Alexa 488-conjugated goat anti-rabbit IgG (H + L) antibody (1:500, A11008; Thermo Fisher Scientific, Carlsbad, CA, USA), rhodamine (TRITC)-conjugated affiniPure goat anti-rabbit IgG (H + L) (1:500; 111-025-003; Jackson ImmunoResearch, West Grove, PA, USA) and biotinylated goat anti-rabbit IgG (1:250; BA-1000; Vector Laboratories, Newark, CA, USA).

For immunofluorescence labeling, tissue sections were washed (PBS, 15 min; 0.2% Triton X-100 in PBS, 30 min; and PBS, 15 min), incubated with blocking solution (5% normal goat serum (S-1000; Vector Laboratories, Newark, CA, USA), 0.2% Triton X-100 in animal-free blocker (SP-5035-100, Vector Laboratories, Newark, CA, USA) for 2 h, then incubated with primary antibody diluted in blocking solution overnight at 4 °C. The sections were washed and then incubated with secondary antibody diluted in blocking solution for 4 h. After washing, the sections were counterstained with 4,6-diaminodino-2-phenylindole (DAPI, 1: 50,000; D1306; Thermo Fisher Scientific, Carlsbad, CA, USA).

For immunoperoxidase labeling, tissue sections were washed, incubated in blocking solution mixed with Avidin D (1:50; Avidin/Biotin Blocking kit; SP-2001; Vector Laboratories, Newark, CA, USA) for 2 h, washed with PBS, then incubated in primary antibody diluted with blocking solution containing biotin (1:50; Avidin/Biotin Blocking kit) overnight at 4 °C. The sections were washed (PBS, 15 min; 0.2% Triton X-100 in PBS, 30 min; and PBS, 15 min), incubated with biotinylated secondary antibody in blocking solution for 4 h, washed, incubated with 0.2% Triton X-100 in PBS containing avidin and biotin complex (1:50 each; Vectastain ABC Elite kit; PK-6100; Vector Laboratories, Newark, CA, USA) for 2 h, washed and then incubated with 3,3-diaminobenzidine (DAB) solution (DAB substrate kit; SK-4100; Vector Laboratories, Newark, CA, USA).

For whole-mount immunoperoxidase labeling, embryos were washed (PBS, 30 min; 0.2% Triton X-100 in PBS, 60 min; and PBS, 30 min), incubated in blocking solution mixed with Avidin D (1:25) for 4 h, washed with PBS, then incubated in primary antibody (1:200) diluted with blocking solution containing biotin (1:25) overnight at 4 °C. Embryos were washed, incubated with biotinylated secondary antibody in blocking solution for 4 h, washed, incubated with 0.2% Triton X-100 in PBS containing avidin and biotin complex (1:25) for 2 h, washed and then treated with DAB solution.

### 2.10. Image Acquisition and Data Analysis

For imaging, transgenic nZRS reporter newts were transferred to a clean Petri dish coated with 3–5 mm of 1% agarose (5091, Takara, Kusatsu, Japan) and positioned under a dissecting microscope (M165 FC; Leica, Tokyo, Japan). Transgenic newts were monitored under a fluorescence filter set for mCherry (exciter: XF1044, 575DF25; emitter: XF3402, 645OM75; Opto Science, Tokyo, Japan). mCherry fluorescence, bright field, and dark field images were captured using a digital camera system (EOS Kiss x7i; Canon, Tokyo, Japan) attached to the microscope and computer. Tissue sections were observed on a fluorescence microscope (BX50; Olympus, Tokyo, Japan) equipped with filter sets for EGFP, mCherry, and DAPI. Images were captured with a charge-coupled device camera system (DP73; cellSens Standard 1.6; Olympus) attached to the BX50 microscope. To visualize thin sections, a laser confocal microscope system (LSM700; ZEN 2009, ver. 6.0.0.303; Carl Zeiss, Oberkochen, Germany) with filter sets for EGFP (Diode 488 Laser; emitter: BP 515–565 nm, Zeiss, Oberkochen, Germany) and mCherry (Diode 555 Laser; emitter: BP 575–640 nm, Zeiss, Oberkochen, Germany) were used.

### 2.11. Digital Software

For plasmid assembly and preparation, SnapGene 7.2.1 was used. Fluorescence, bright-light image contrast, brightness, and sharpness were adjusted using Photoshop 21.2.0 (Adobe, San Jose, CA, USA). Figures were prepared using Illustrator 27.4.1 graphics software (Adobe).

## 3. Results

### 3.1. Characterization of the Newt nZRS Enhancer

**Figure 1:**
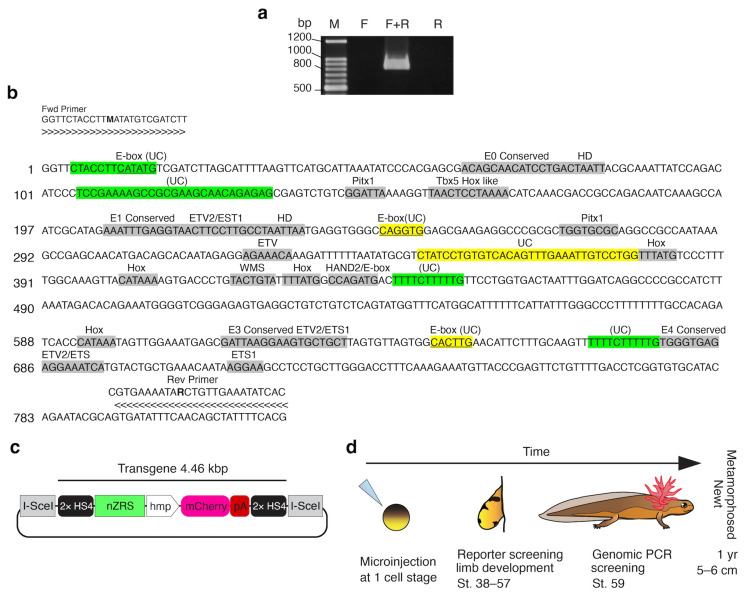
Generation of transgenic nZRS reporter newt. (**a**) PCR amplification of the newt (*C. pyrrhogaster*) ZRS enhancer from genomic DNA. F: forward degenerate primer; R: reverse degenerate primer. To rule out nonspecific PCR amplicons using degenerate primers, PCR reactions using a single primer (lane F or lane R) were used as controls. (**b**) Nucleotide sequence of the newt ZRS sequence, 819 bp (accession no. PP691624). Characterization of mouse binding sites identified in the newt nZRS in grey, vertebrate uncharacterized (UC) conserved sites in yellow, and urodele uncharacterized sites in green. Vertebrate ZRS alignments are available in Tables S1–S8 and Figure S3. Forward and reverse degenerate primer sets are shown on both ends of the sequence. Degenerate nucleotides indicated with bold. (**c**) Illustration of the transgene nZRS reporter plasmid. hmp: human minimal promoter; HS4: Chicken single core insulator. (**d**) Schematic summary of nZRS reporter transgenesis and screening.

We isolated the newt (*C. pyrrhogaster*) nZRS fragment (819 bp) from genomic DNA, and a single PCR product was observed (Figure 1a). The nZRS fragment was sequenced (Figure 1b) and is available at Genbank with accession number PP691624. Next, we confirmed that the newt nZRS enhancer sequence contained DNA binding sites to drive reporter expression. Therefore, the nZRS sequence was characterized with previous mouse-conserved DNA-protein binding sites, conserved uncharacterized sites, and urodele uncharacterized sites (Figure 1b). MEME Suite was used to produce aligned conserved sequence blocks with other known vertebrate ZRS sequence regions (Tables S1–S8). Similar to the mouse ZRS, nZRS contains ETS (erythroblast transformation-specific) conserved sites E0, E1 (containing a snake-specific deletion site), E3, and E4 (Figure 1b; Tables S1, S3, S7, and S8), but devoid of the E2 site (Figure S3) [20,37]. EST sites are known key regulators of Shh positional expression in the limb [20,37]. The nZRS contains two ETV2 sites (ACTTCCTT and AAGGAAGT). ETV2, a master regulator of hematoendothelial lineages, was recently shown to be an upstream regulator of Shh expression, chromatin regulation, and transcriptional activation during limb bud development [38]. In the context of tissue regeneration, ETV2 can induce vascular regeneration following murine heart injury in vivo [39]. Four Hox sites, WMS (Werner mesomelic syndrome, a point mutation site), and HAND2/Ebox [40,41] were also identified in nZRS (Figure 1b; Table S6). HAND2 is a known regulator of Shh in the limb bud mesenchyme [40]. Two Pitx1 sites (GGATTA and TGGTGCGC) that modulate hindlimb expression [42] were identified in the nZRS sequence. Two vertebrate uncharacterized conserved regions were also identified: an Ebox site and a 32-bp box (Figure 1b; Tables S3 and S5). Unique uncharacterized regions of nZRS were identified using Pro-Coffee alignment, providing a heat map of regions with low-high homology (Figure S3). Beyond development, adult newts regulate Shh expression during limb regeneration [19]. Therefore, we examined potential unique sites that may explain Shh regulation beyond metamorphosis. We identified the following urodele sites: two copies of the TTTTCTTTTTG sequence found in newt ZRS (*C. pyrrhogaster*, *C. orientalis*, and *P. waltl*), an upstream sequence containing an Ebox site (CTACCTTCATATG), and TCCGAAAAGCCGCGAAGCAACAGAGAGCG (Figure 1b). Since the ZRS region is sensitive to even single point mutations associated with human disease [41], it would be interesting to examine if these urodele sites have any functional binding sites, a subject for future research.

### 3.2. Functional Analysis of the nZRS Enhancer

**Figure 2:**
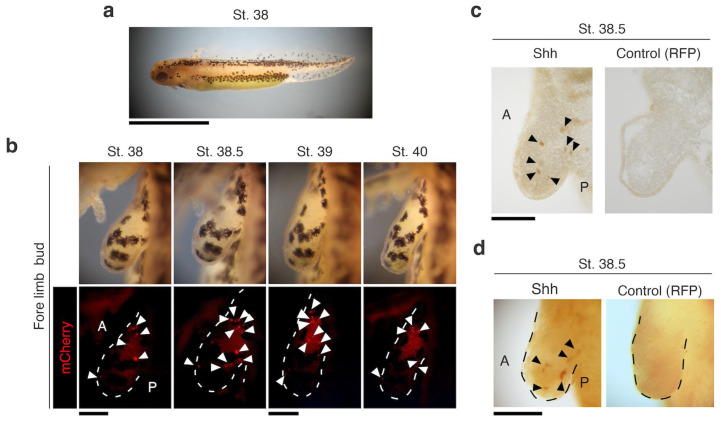
nZRS-reporter expression during forelimb bud development. (**a**) Representative ZRS reporter expression at the larval stage (st.) 38 (lateral view; *n* = 6 larval newts). (**b**) Using larvae from (**a**), mCherry+ cells were detected in the newt limb bud (dorsal view) anterior-posterior at st. 38, and monitored to st. 40. White arrowheads indicating mCherry+ cells. (**c**) Anti-Shh immunoreactivity in the wild-type developing limb bud at st. 38.5 (*n* = 3, for each), the ABC-DAB method was used. In the wild-type samples, RFP (anti-RFP antibody rabbit polyclonal) was used as the negative control. Black arrowheads indicate a few Shh+ cells. (**d**) Whole mount Shh staining at st. 38.5 (*n* = 3, for each), with similar staining used in (**c**). Black and white dashed lines indicated limb bud. A: anterior; P: posterior. Scale bars: (**a**), 5 mm; (**b**–**d**), 250 µm.

**Figure 3:**
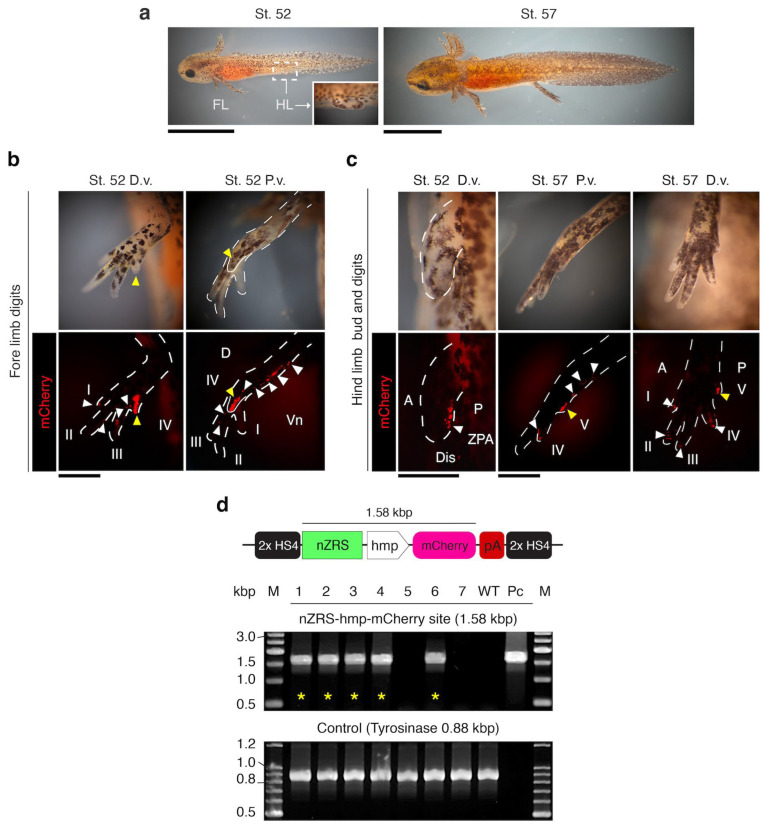
nZRS reporter expression during hind limb bud and digit development. (**a**) Representative nZRS reporter expression at larval stages (st.) 52–57 (lateral view; *n* = 6). Inset showing the hind limb bud. (**b**) At st. 52, mCherry reporter signal was detected in digits I–IV, with IV having a noticeable expression. A stream of mCherry+ cells can be observed along the posterior-ventral region from the proximal elbow to digit IV. (**c**) St. 52 also exhibited marked expression of the hind limb bud, where mCherry+ cells were localized in the posterior region of the hind limb bud, with a ZPA-like pattern. At st. 57, mCherry+ cells were located along the posterior limb in digits I–V, with digit V having noticeable expression. White arrowheads indicate mCherry+ cells. Yellow arrowheads indicate digit IV (forelimb) or V (hindlimb) with mCherry signal. (**d**) Representative screening by genomic PCR at larval stages 57–59 before metamorphosis (Table S9). Lane numbers indicate the transgenic nZRS reporter individuals (*n* = 7). Individuals with the complete transgene (1.58 kbp) are indicated with a yellow asterisk (*n* = 5/7). A 0.88 kbp region of the tyrosinase gene was used as the positive control. A: anterior; D: dorsal; D.v.: dorsal view; Dis: distal; FL: forelimb; HL: hindlimb; M: size marker; P: posterior; Pc: plasmid DNA of pnZRS-hmp-mcherry01 (positive control); P.v.: posterior view; WT: wild-type genomic DNA (negative control); Vn: ventral. Scale bars: (**a**), 5 mm; (**b**,**c**), 0.5 mm.

For functional analysis of the nZRS sequence, we developed the plasmid pnZRS-hmp-mcherry01 (Figure 1c,d) and used it for F0 transgenic newts (Table S9). Reporter expression was detected at larval stage 38 in the limb bud (Figure 2a,b; *n* = 6). To confirm the expression of the Shh protein, we developed a newt anti-Shh rabbit polyclonal antibody and tested for specificity in known Shh-expressing tissue, the retina (Figure S1). We detected Shh protein localization in newt retinal ganglion cells, as was previously described in fish [27] (Figure S1c). In the WT limb bud stage 38.5, Shh protein expression coincided with nZRS reporter expression (Figure 2c,d). We further validated the nZRS reporter expression at other development stages and in the hindlimb. The newt nZRS reporter was detected in the hindlimb bud and digit patterning (Figure 3), suggesting that this nZRS reporter possessed spatial and temporal regulation during limb development. Unlike development in the mouse limb, where the fore limb and hind limbs develop almost in synchrony, in urodele amphibians, the fore limb with four digits develops first, followed by the hind limbs with five digits. During development, newt larval digits are patterned with a pre-axial dominance [2,22]. Here, the nZRS reporter was observed during forelimb digit formation at stage 52, with reporter expression localized with higher intensity in the fourth digit along the posterior-ventral limb region (Figure 3b). At stage 52, during hindlimb bud development, a ZPA-like cluster of mCherry cells polarized in the posterior (Figure 3c). At stage 57, the fifth digit of the hindlimb also expressed mCherry at the posterior-ventral limb region (Figure 3c). Reporter mCherry+ cells could be tracked above the elbow/knee region (Figure 3b,c). Transgenic nZRS reporter newts having the phenotype described in Figure 2 and Figure 3 were further screened by genomic PCR (Figure 3d) to ensure that the full functional transgene region (1.58 kbp) was inserted. Individuals without the functional transgene region were considered negative individuals and later separated.

### 3.3. nZRS Reporter Activity Following Metamorphosed Limb Regeneration

**Figure 4:**
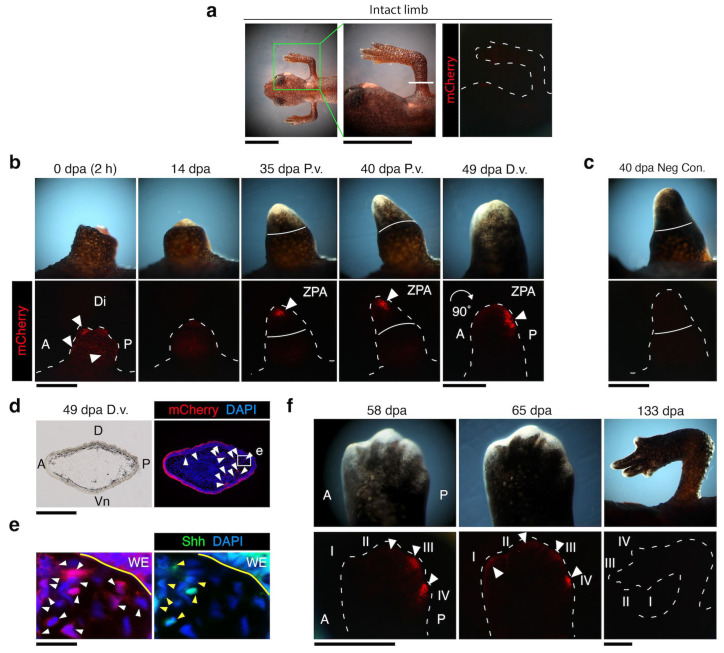
nZRS reporter expression during metamorphosed limb regeneration and autopod patterning. (**a**) Representative transgenic nZRS reporter newt with an intact limb (dorsal view), *n* = 9. The white line indicates the mid humerus (stylopod) amputation plane. (**b**) Following limb amputations, the mCherry reporter signal (white arrowheads) was detected at 2 h post-amputation in the anterior region. At 14 dpa, the reporter signal was not detected. However, during mid-blastema, at 35 dpa, a defined cluster of cells was detected. Solid white line indicates the amputation plane. At 40 dpa, a mCherry+ cluster was localized in the posterior of the late-blastema, ZPA-l. At the palette stage, at 49 dpa, just before digit I regeneration, a mCherry+ cluster was polarized in the posterior region. (**c**) Negative control, wild-type individuals uninjected with reporter plasmid, *n* = 3 limbs. (**d**) Cross section of transgenic ZPA region at 49 dpa, *n* = 3. (**e**) Magnification of white box (**d**) indicating overlap between mCherry+ and Shh+ cells. The yellow line indicates the margin of the WE. White arrowheads indicate mCherry+ cells; yellow arrowheads indicate Shh+ cells. (**f**) At 58 dpa, reporter expression was localized in digits III and IV. By 65 dpa, a weak signal was detected in the anterior digits I to III, and digit IV showed a reduction of reporter expression. White dashed lines indicates contour of the limb.. A: anterior; D: dorsal; Di: distal; dpa: days post-amputation; D.v.: dorsal view; P: posterior; P.v: posterior view; Vn: ventral; ZPA: zone of polarizing activity; WE: wound epithelium. Scale bars: (**a**), 0.5 cm; (**b**,**c**,**f**), 1 mm; (**d**), 0.5 mm; and (**e**), 50 µm.

Transgenic nZRS reporter newts (described in Figure 2 and Figure 3, with positive functional transgene insertions) were reared beyond metamorphosis. When newts were one year old (with a body length of 5.5–6 cm), terrestrial metamorphosed limb regeneration was examined, *n* = 9 (Figure 4). No mCherry reporter was detected in the intact limb prior to limb amputations (Figure 4a). Shh protein expression in the intact limbs of adult WT newts was also not detected at mid-stylopod or zeugopod (Figure S2a,b). Next, we amputated the limb of nZRS transgenic newts at the mid-stylopod (humerus) and tracked the mCherry reporter during regeneration. In transgenic nZRS reporter newts, a signal was observed as early as 2 h following amputation and not detected at 14 days post-amputation (dpa) (Figure 4b). At 35 dpa, the nZRS reporter showed mCherry+ cells clustering in the blastema, displaying signs of early ZPA organization (Figure 4b). Note that the adult WT blastema displayed Shh protein expression at 30 dpa (Figure S2c). By 40 dpa, the nZRS reporter was localized in the posterior blastema (Figure 4b).

To rule out autofluorescence, we used non-injected embryos (control, WT) from the same clutch, reared in parallel with the transgenic newts of the same age (Table S9). Non-injection newts of the same age did not show any reporter signal (Figure 4c). From 40–49 dpa, there was a 90° posterior tilt between the regenerating blastema and the base of the limb. At the late blastema to early palette stage, at 49 dpa, just before the regeneration of digits, the blastemal ZPA became polarized in the posterior region in preparation for digit regeneration (Figure 4b,d,e). At 58 dpa, digit regeneration and cartilage condensation were observed, where digits III and IV displayed a reporter signal (Figure 4f). However, a decreasing signal at 65 dpa was observed in digit IV, while a weak signal was detected in digits I–III (Figure 4f). By 133 dpa, the nZRS reporter signal was not detected, and pigmentation had increased throughout the regenerating digits.

### 3.4. nZRS Reporter Overlaps with Shh Protein Expression

**Figure 5:**
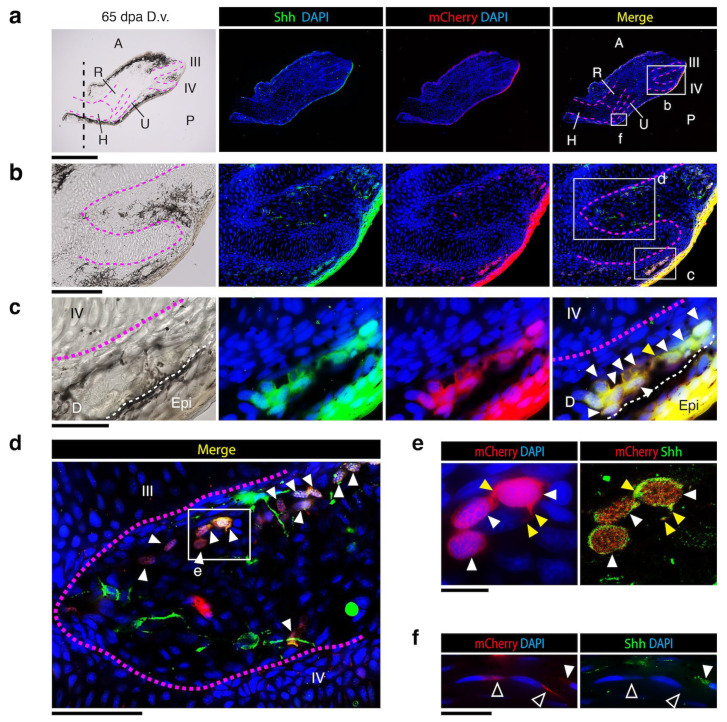
Localization of Shh expression overlaps with transgenic nZRS mCherry reporter cells. (**a**) Representative regenerating limb at 65 dpa (Figure 4) of transgenic nZRS reporter newt, *n* = 3 limbs. The amputation plane is indicated by a black vertical dashed line. Sections were stained with anti-Shh rabbit polyclonal and anti-RFP mouse monoclonal antibodies. (**b**) Magnification of (**a**) showing digits III and IV. (**c**) Magnification of box in (**b**) showing the distal posterior region of digit IV. Here, double-labeled cells with Shh+ and mCherry+ were localized in the regenerating dermal layer. A white dotted line indicates the margin between the dermis and epidermis. (**d**) Magnification of box in (**b**) showing inter-digit region between digits III and IV. (**e**) Magnification of box in (**d**) showing localization of mCherry in the cytoplasm (left). The same cells were imaged using a laser confocal microscope, showing the localization of Shh in the membrane (right) using thin optical sections (1.3–2.0 µm thick). Yellow arrowhead indicates a protruding cytoplasm (**c**,**e**). (**f**) Magnification of box in (**b**) showing proximal posterior region of digit IV. Double-labeled cells can be seen along the surrounding dermis of regenerating digits. Dotted pink lines indicate the margins of regenerating skeletal tissue. White arrowheads indicate Shh+/mCherry+ cells; open arrowheads indicate Shh−/mCherry+ cells. A: anterior; D: dermis; dpa: days post-amputation; D.v.: dorsal view; Epi: epidermis; H: humerus; P: posterior; R: radius; U: ulna. Scale bars: (**a**), 1 mm; (**b**), 200 µm; (**c**,**f**), 50 µm; (**d**), 100 µm; and (**e**), 20 µm.

**Figure 6:**
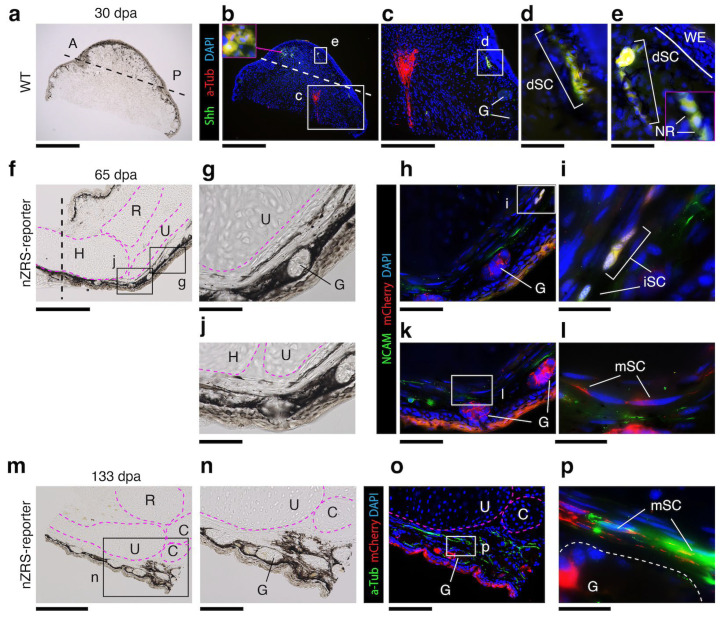
nZRS reporter system during metamorphosed limb regeneration. (**a**) The wild-type adult blastema section at 30 dpa (*n* = 3); black dotted line indicates amputation site. (**b**) The section in (**a**) stained with anti-acetylated tubulin mouse monoclonal and anti-Shh rabbit polyclonal antibodies. The amputation plane is indicated by a white dotted line. Here, cutaneous nerve fiber Schwann cells (SCs) undergo distal migration and blastema cells expressing Shh+/a-tubulin+ (shown in pink inset). (**c**) Magnification of whited boxed region in (**b**) noncutaneous SCs did not express Shh but expressed acetylated tubulin. (**d**) Magnification of white boxed region in (**c**) showing cutaneous dedifferentiating SCs (dSCs) expressing Shh+/a-tubulin+ (indicated by white brackets) were found below the amputation site). (**e**) Magnification of (**b**) showing dSCs contributing to the limb blastema, migrating towards the WE margin, indicated by a solid white line. Pink inset showing dSCs (white brackets) with NR. (**f**) Transgenic nZRS reporter new limb section at 65 dpa, amputated at the mid humerus (indicated by a black vertical dotted line). (**g**) Magnification of the boxed region in (**f**), showing the posterior regenerated elbow, and glands can be seen in the maturing skin. (**h**) Sections were stained with anti-NCAM rabbit polyclonal and anti-RFP mouse monoclonal antibodies. Glands expressed weak mCherry but not NCAM. (**i**) Magnification of box in (**h**): here, immature SCs (iSCs) expressed mCherry+/NCAM+ located distal to the amputation site (indicated by white brackets). (**j**) Magnification of boxed region in (**f**); this region has a closer proximity and maturity than (**g**). (**k**) Sections were stained in a manner similar to (**h**). (**l**) Magnification of boxed region (**k**) where mSCs expressed mCherry but not NCAM. (**m**) Transgenic nZRS reporter new limb section at 133 dpa, showing the posterior regenerating wrist joint. (**n**) Magnification of boxed region in (**m**) with a maturing gland. (**o**) Sections were stained with anti-acetylated tubulin mouse monoclonal and anti-RFP rabbit polyclonal antibodies. (**p**) Magnification of boxed region in (**o**): here, mSCs are expressing mCherry+/acetylated-tubulin+. Pink dashed lines indicate regenerating skeletal features. White dashed lines indicate skin gland. A: anterior; C: carpal bones; dpa: days post-amputation; G: gland; H: humerus; NR: nodes of Ranvier; P: posterior; R: radius; U: ulna; WE: wound epithelium. Scale bars: (**a**,**b**), 1mm; (**c**,**f**,**m**), 500 µm; (**g**,**h**,**j**,**k**,**n**,**o**), 200 µm; (**d**,**e**), 100 µm; and (**i**,**l**,**p**), 50 µm.

To identify the nZRS reporter mCherry+ and Shh+ cells observed in the blastemal ZPA (Figure 4e), we collected limb samples at 65 dpa (*n* = 3 limbs) (Figure 4f) and prepared sections for IHC. This day provided immature to mature regenerating tissue along the proximal to distal axis (Figure 5a). Here, we screened tissue sections to confirm if our nZRS reporter newts had overlapping Shh protein expression in cells. Sections were stained with anti-Shh rabbit polyclonal and anti-RFP mouse monoclonal antibodies (Figure 5). At 65 dpa, in transgenic nZRS reporter newts, skeletal condensation and bone maturation were observed in the humerus, ulna, radius, and digit patterning (Figure 4f and Figure 5a). We examined our nZRS reporter for the mCherry signal in regions distal (Figure 5c–e) and proximal (Figure 5f) to the amputation plane at the mid humerus (Figure 5a). Here, with nZRS reporter newts, we identified and co-localized mCherry+ and Shh+ cells in the dermal region of the regenerating limb (Figure 5c–f). In the distal region, in a few cells expressing Shh+/mCherry+, the cytoplasm had protruding structures, *n* = 1/9 (Figure 5c) and *n* = 1/3 (Figure 5e). mCherry protein expression was identified in the cytoplasm, giving the appearance of a webbed nucleus (Figure 4e and Figure 5e). Shh protein expression was co-localized in the cytoplasm and cell membrane (Figure 5e). Previously, newts expressing mCherry and acetylated tubulin in Schwann cells (SCs) gave a similar webbed nuclear appearance with a flattened morphology [8]. In the adult WT at 14 and 30 dpa, Shh expression was detected in myelinating Schwann cells (mSCs) of cutaneous nerves adjacent to glands (Figure S2c–i). Shh+/mCherry+ cells of the nZRS reporter were commonly observed in group formation along the regenerating dermis (Figure 5c–f). In proximal regions of the limb where the tissue had regenerated, weak expression of mCherry+/Shh− or mCherry+/Shh+ cells were detected, and these cells had a flattened SC-like morphology (Figure 5f), similar to a previous study [8]. We reasoned that these distal Shh+/mCherry+ cells were probably immature SCs (iSCs) or dedifferentiated blastemal cells (Figure 4e) derived from mSCs observed in adult limb regeneration at 14dpa, 30 dpa (Figure S2) and proximal 65 dpa (Figure 5f). We, therefore, applied the nZRS reporter with other known SC markers that could provide evidence for the involvement of SCs in our system (Figure 6).

### 3.5. Cutaneous Regenerating Schwann Cells and Glands Express Shh

We revisited which mSCs were actually contributing to the blastema. Here, we co-labeled 30 dpa blastema’s of WT limbs with anti-Shh and anti-Acetylated tubulin, *n* = 3 adult blastema. In the newt, we previously showed that mSCs express acetylated tubulin [8]. Here, we found that Shh+/acetylated tubulin+ SCs of the dermis, but not Shh−/acetylated tubulin+ SCs, were participating in the blastema (Figure 6a–e and Figure S4). At 30 dpa, Shh+ SCs cells appear to be dedifferentiating and change morphology (Figure 6d,e, Figures S2 and S4). We used our nZRS reporter that aligned with Shh protein expression (Figure 5) to examine another marker of SCs, neural adhesion molecule 1 (NCAM) [43]. NCAM, also known as CD56, is commonly expressed in immature, sensory, and non-myelinating SCs [43,44,45]. NCAM has also been reported in mesenchymal cells of the newt blastema [46]. In nZRS reporter newts, iSCs expressed mCherry+/NCAM+, but maturing mCherry+ mSCs with a flattened morphology did not express NCAM at 65 dpa (Figure 6f–l). At 133 dpa, mSCs with residual mCherry reporter protein expressed acetylated tubulin (Figure 6m–p) but not Shh, as observed at 65 dpa (Figure 5f). Dermal glands in the regenerating limb expressed Shh protein (Figure S2c–i) and the nZRS mCherry reporter (Figure 6f–p).

## 4. Discussion

The ZRS is an important regulatory sequence for the proper expression of Shh in the developing ZPA in the limb bud [20,21,37,41]. The ZRS is finely tuned and is sensitive to mutations. In humans, point mutations in the ZRS can lead to congenital abnormalities called “ZRS-associated syndromes” such as preaxial polydactyly type 2, triphalangeal thumb polysyndactyly, syndactyly type 4, and Werner mesomelic syndrome (WMS) [21,37,41]. When considering newt-like regeneration strategies for medical applications, an important aspect to address is how regenerating cells can pattern correctly in adults. Here, we used the terrestrial newt as a potential model organism to study adult cells capable of regulating the ZRS. The SC, the most common cell type in the peripheral nervous system, originates from the neural crest during development [47]. These cells, also known as peripheral nerve neuroglia, are found in both the peripheral and autonomic nervous systems [47,48]. The ability of peripheral nerves to regenerate after mammalian injury relies heavily on SCs, with the rat being the most noticeable [49,50]. Following injury, SCs undergo significant reprogramming, transitioning from their roles as myelinating or non-myelinating (Remak) cells to repair-specific functions [45,51]. In their repair state, SCs clear myelin debris, release signals to attract macrophages, support neuronal regrowth, form axon guidance pathways known as Büngner bands, and remyelinate the axon [45,52]. Interestingly, during nerve injury, SCs not only dedifferentiate but have an altered phenotype during the repair state, activating a unique gene expression program that differs from other stages of their development [45,49]. For example, lineage tracing experiments found no Shh expression from neural crest cells to mature SCs [53]. However, Shh was highly induced in SCs following nerve injury and was regulated by c-JUN [49,54]. Rodent SCs activate Shh expression with unique injury enhancers [48]. In serum-free culture conditions, adult rat sciatic and palatal myelinating SCs respond to FGF-2 and EGF, forming neurospheres that upregulate the pluripotency factors Sox2, Klf4, c-Myc, and Oct4, the NF-kB subunits p65 and p50, and the NF-kB inhibitor IkB-b [55].

During terrestrial newt limb regeneration, SCs are capable of self-regenerating [8]; they contribute to blastema formation (a dedifferentiation state) [56] and express nAG for the proximal/distal Prod1/nAG signaling system [14,15]. During the adult blastemal stage, the pluripotency factors Sox2, Klf4, and c-Myc are expressed [57]. Following the formation of the blastema, to successfully regenerate the missing limb and digits, adult newts reactivate Shh in the blastema for proper patterning [19]. Here, using the endogenous newt Shh limb enhancer, nZRS, we developed a new reporter system for the newt *C. pyrrhogaster*. We demonstrate that the nZRS-mCherry reporter expression forms a “blastemal ZPA” reminiscent of the developing vertebrate limb bud [17,18,19,20,40]. When we examined cells capable of driving our nZRS-reporter, we found overlapping expression with Shh, NCAM (non-myelinating), and acetylated tubulin (myelinating), known markers of SCs [8,43,44,45]. Following limb amputation, Shh was expressed in subcutaneous mSCs. Newt glands during regeneration were also shown to drive the nZRS-reporter and express Shh protein. This suggests that newt glands and associated SCs innervating the skin may participate in patterning the adult limb. In support of this, in a previous study, we found that grafting intact skin followed by amputations altered patterning with supernumerary digits (dorsal-dorsal grafts) or mirror regenerates (posterior-posterior grafts) were observed [23]. It would be interesting to study whether a similar partnership between glands and SCs exists in the anterior-posterior patterning, as shown in the proximal/distal Prod1/nAG signaling system [14,15]. Here, our findings contribute to identifying SCs as a potential cellular source of Shh for limb patterning in the newt. Using the nZRS reporter system in combination with an anti-Shh antibody, we observed that SCs are capable of reporter transcription and Shh protein expression. Further research is needed to examine other genes involved in the Shh signaling pathway in this system. However, it is now possible to envision the development of genetic manipulation studies using this new nZRS reporter system, such as functional gene analysis targeting SCs. It would be interesting to consider comparing murine repair SCs with newt blastemal SCs, thus providing insight into molecular and cellular mechanisms used by SCs following injury. Newts have a strong regenerative potential; newt SCs are an excellent model following nerve injuries that can provide novel insights into regenerative medicine. The nZRS reporter system can be used in basic medical research to compare murine SCs following injury.

## Data Availability

All data used in this study are available from the corresponding authors upon reasonable request.

## Supplementary Materials

The following supporting information can be downloaded at https://www.mdpi.com/article/10.3390/biomedicines12112505/s1, Figure S1. Production of the newt anti-Shh antibody and validation. Figure S2. Shh protein detection using IHC with anti-Shh polyclonal antibody (Figure S1) was observed in the regenerating limb (dermal nerve mSCs and glands) but not in the intact limb. Figure S3. Vertebrate ZRS alignment. Figure S4. Expression of acetylated-tubulin and Shh protein expression in adult wild type blastema at 30 dpa. Table S1. Conserved DNA motif/binding sites of the newt nZRS enhancer aligned with those of other vertebrates. Table S2. Conserved DNA motif/binding sites of the newt nZRS enhancer, detected after alignment with those of other vertebrates. Table S3. Conserved DNA motif/binding sites of the newt nZRS enhancer aligned with those of other vertebrates. Table S4. Conserved DNA motif/binding sites of the newt ZRS enhancer aligned with those of other vertebrates. Table S5. Conserved uncharacterized DNA site of the newt nZRS enhancer aligned with that of other vertebrates. Table S6. Conserved DNA motif/binding sites of the newt nZRS enhancer aligned with those of other vertebrates. Table S7. Conserved DNA motif/binding sites of the newt nZRS enhancer aligned with those of other vertebrates. Table S8. Conserved DNA motif/binding sites of the newt nZRS enhancer aligned with those of other vertebrates. Table S9. Generation of transgenic nZRS reporter newts at F0 [13,20,27,37,38,39,40,41,58,59].
