# Supplementary material for: Development of a ZRS Reporter System for the Newt (Cynops pyrrhogaster) During Terrestrial Limb Regeneration
Source: Biomedicines. 2024 Nov 1;12(11):2505. doi: 10.3390/biomedicines12112505 (PMC11591917; doi:10.3390/biomedicines12112505)
Supplement: Supplementary file 1 [file biomedicines-12-02505-s001.zip › biomedicines-3284061-supplementary.pdf]

Supplementary Information

for

Development of a ZRS Reporter System for the Newt (*Cynops pyrrhogaster*) During Terrestrial Limb Regeneration

1. Supplementary Figures
2. Supplementary Tables

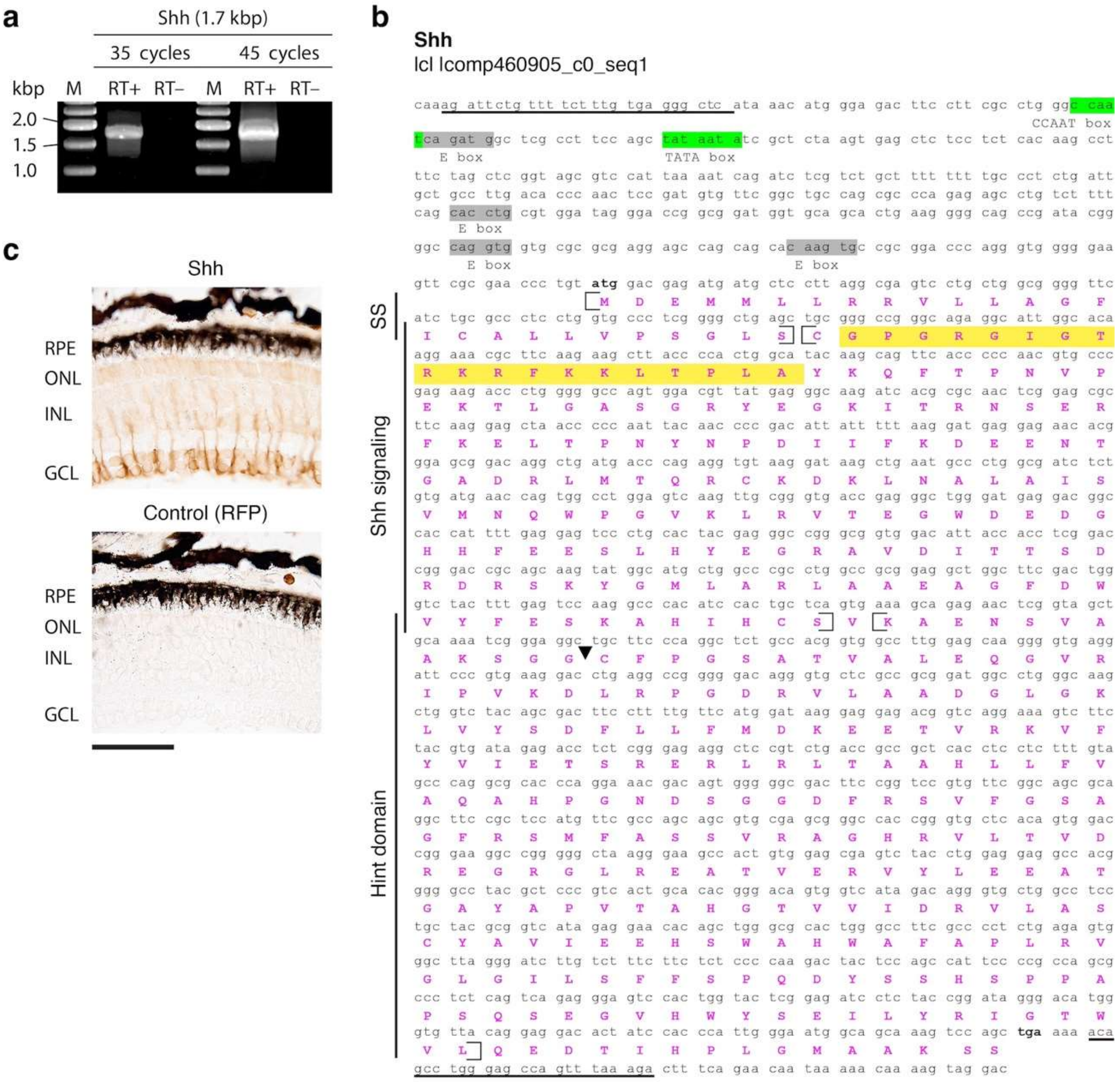

Figure S1. Production of the newt anti-Shh antibody and validation. (**a**) PCR for Shh using limb blastema cDNA. Increasing the number of cycles from 35 to 45 did not result in the detection of additional amplicons. In each sample, the same template was used. +: template with reverse transcription. –: template without reverse transcription. M: marker. (**b**) *Cynops pyrrhogaster* cDNA sequence of Shh (**a**) was referenced to a Shh transcript (comp460905_c0_seq1) found in the transcriptome database TOTAL [13]. Underlined are the primer sets for PCR to amplify the Shh transcript (**a**). The Shh nucleotide sequence is available at Genbank with accession number PQ306330. The 5′ UTR sequence contains the CCAT box and the TATA box (highlighted in green) previously described in the human Shh gene [58]. Three uncharacterized E-boxes were found in the 5′ UTR, highlighted in grey. The amino acid sequence is shown in pink capital letters. The amino acid sequence highlighted in yellow indicates the epitope site for antibody production. The black arrowhead indicates the conserved site for internal auto-proteolytic cleavage of Shh, described previously [59]. Open and closed brackets indicate amino acids corresponding to domains on the left. (**c**) We validated our antibody with IHC. The ABC-DAB method was used to detect anti-Shh immunoreactivity in the wild type newt retina, n=3 eyeballs. Anti-RFP antibody was used as a negative control, n=3 eyeballs. Shh was detected in retinal ganglion cells as previously described in the fish retina [27,59]. GCL: ganglion cell layer; INL: inner nuclear layer; ONL: outer nuclear layer; RPE: retinal pigmented epithelium. Scale bar: (**c**), 100 µm.

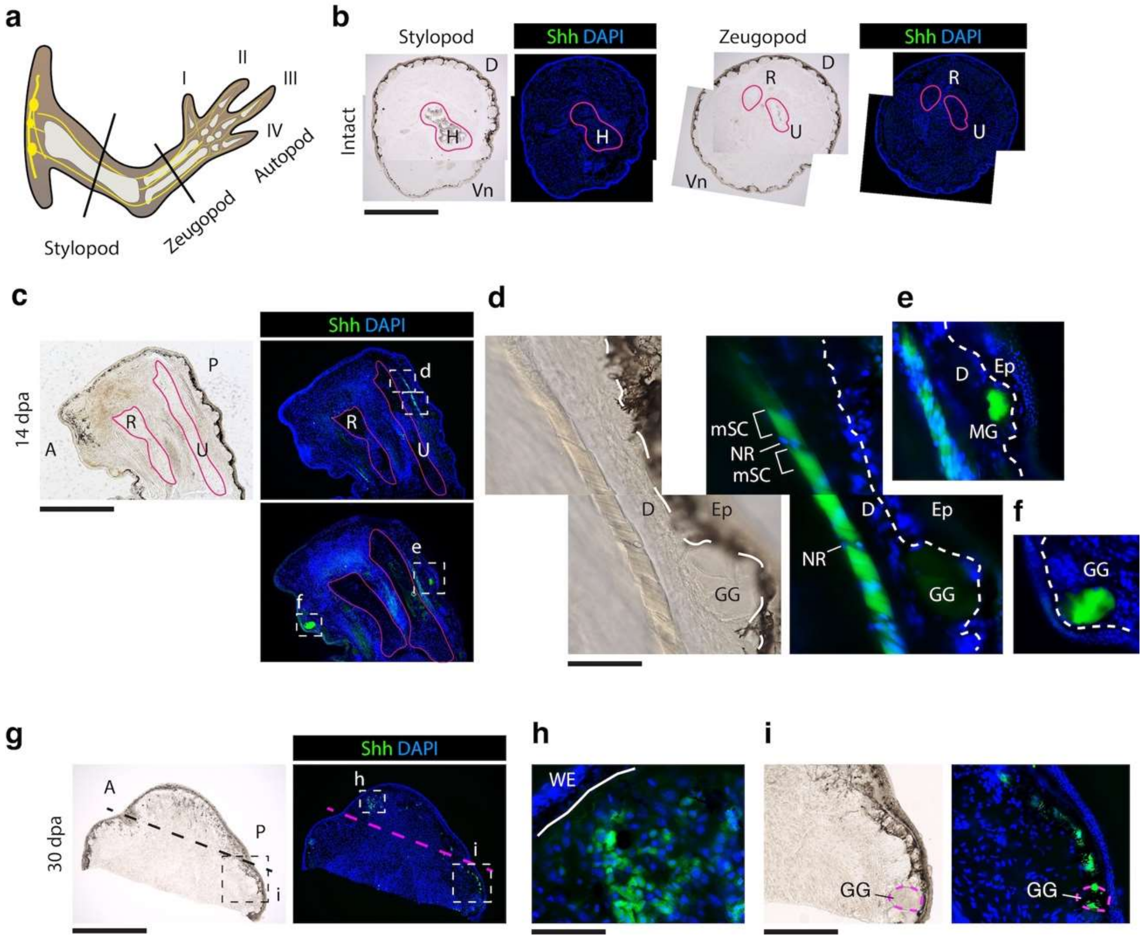

Figure S2. Shh protein detection using IHC with anti-Shh polyclonal antibody (Figure S1) was observed in the regenerating limb (dermal nerve mSCs and glands) but not in the intact limb. (**a**) Illustration of the adult newt limb, black solid lines indicating amputations sites used to examine Shh protein in intact limbs. (**b**) Cross-sections of intact limbs were prepared at the level of mid stylopod (humerus) and mid zeugopod (ulna, radius) for IHC, n=3 limbs. Shh protein expression was not detected. Pink outline indicates bone. (**c**) Adult limb, following 14 dpa with Shh expression in the dermal nerve, posterior to the ulna, and below the amputation site. Images to the right obtained from (**c**) and stained with IHC. (**d**) High magnification of the boxed region in (**c**). Image to the right, IHC using anti-Shh antibody; here, the Shh nerve fiber shows myelination and nodes of Ranvier. Dotted line indicates the margin between the dermis and epidermis. (**e**) High magnification of the boxed region in (**c**). Here a posterior dermal gland and the dermal nerve fiber expressed Shh. Note the hyper nucleation surrounding the nerve fiber, and early demyelination. (**f**) High magnification of the boxed region in (**c**). An anterior granular gland expressing Shh. (**g**) Adult limb blastema at 30 dpa. Amputation plane indicated by a black dotted line. Image to the right, IHC. Amputation plane indicated by a pink dotted line. (**h**) High magnification of the boxed region in (**g**) showing expression of Shh blastemal cells below the WE. White solid line indicating the WE margin. (**i**) High magnification of the boxed region in (**g**) showing expression of Shh dermal nerve fiber and a posterior gland below the site of amputation (also observed in Figure 6a-e). A: anterior; D: dorsal; dpa: days post amputation; Ep: epithelium; GG: granular gland; H: humerus; MG: mucosal gland; mSC: myelinated Schwann cell; NR: nodes of Ranvier; P: posterior; R: radius; U: ulna; WE: wound epithelium. Scale bars: (**b**), 2 mm; (**c**, **g**), 1mm; (**d**, **e**, **f**, **i**), 200µm; and (**h**), 100µm.

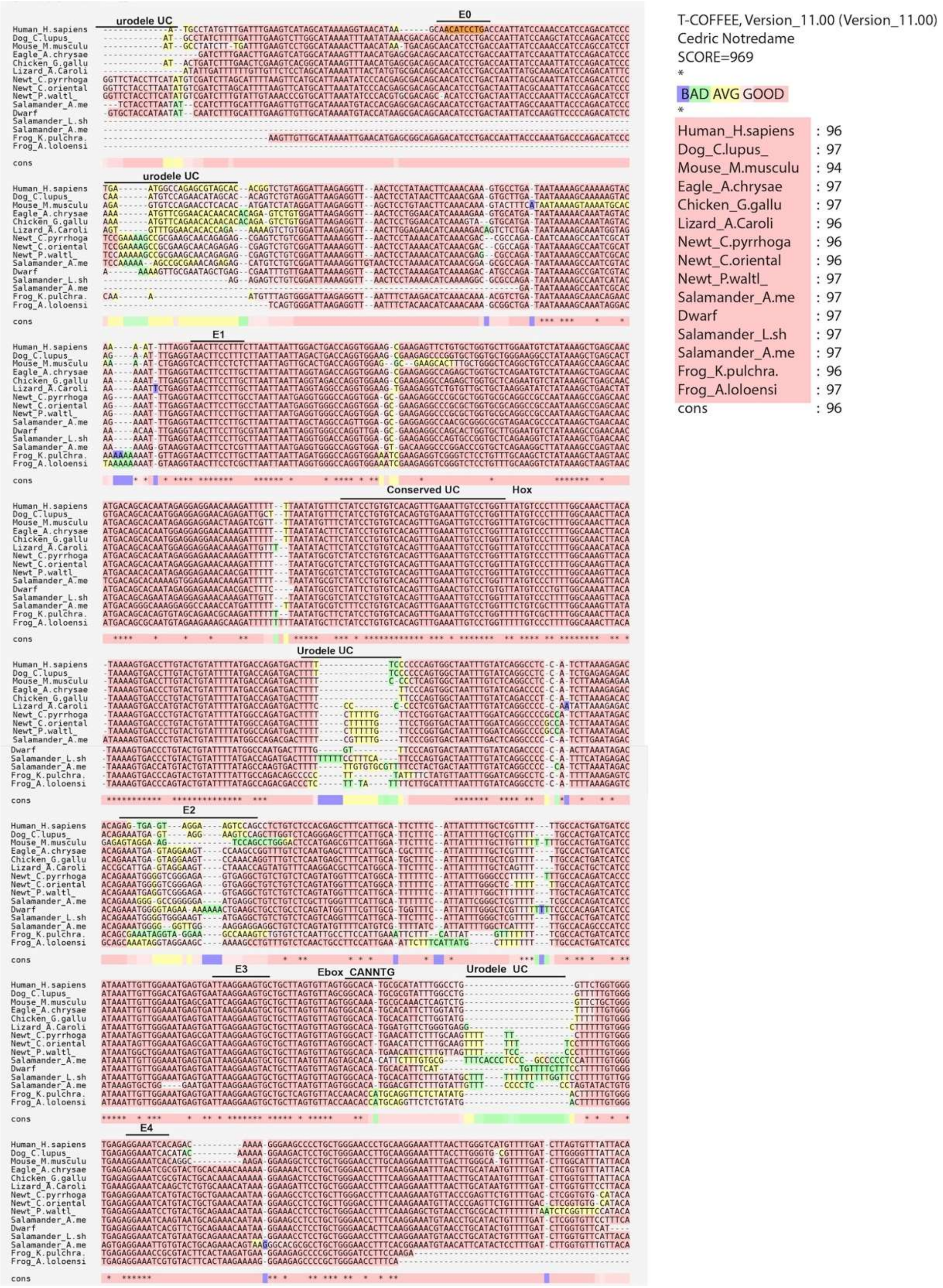

Figure S3. Vertebrate ZRS alignment.

Mouse ETS sites are included as E0-E4 [20]. Here we show regions with unique sequences among urodele species that are urodele uncharacterized (UC) and conserved regions among other vertebrates. We detected a 32-bp vertebrate conserved uncharacterized site (UC) (Table S5) upstream of a reported mouse Hox site [40].

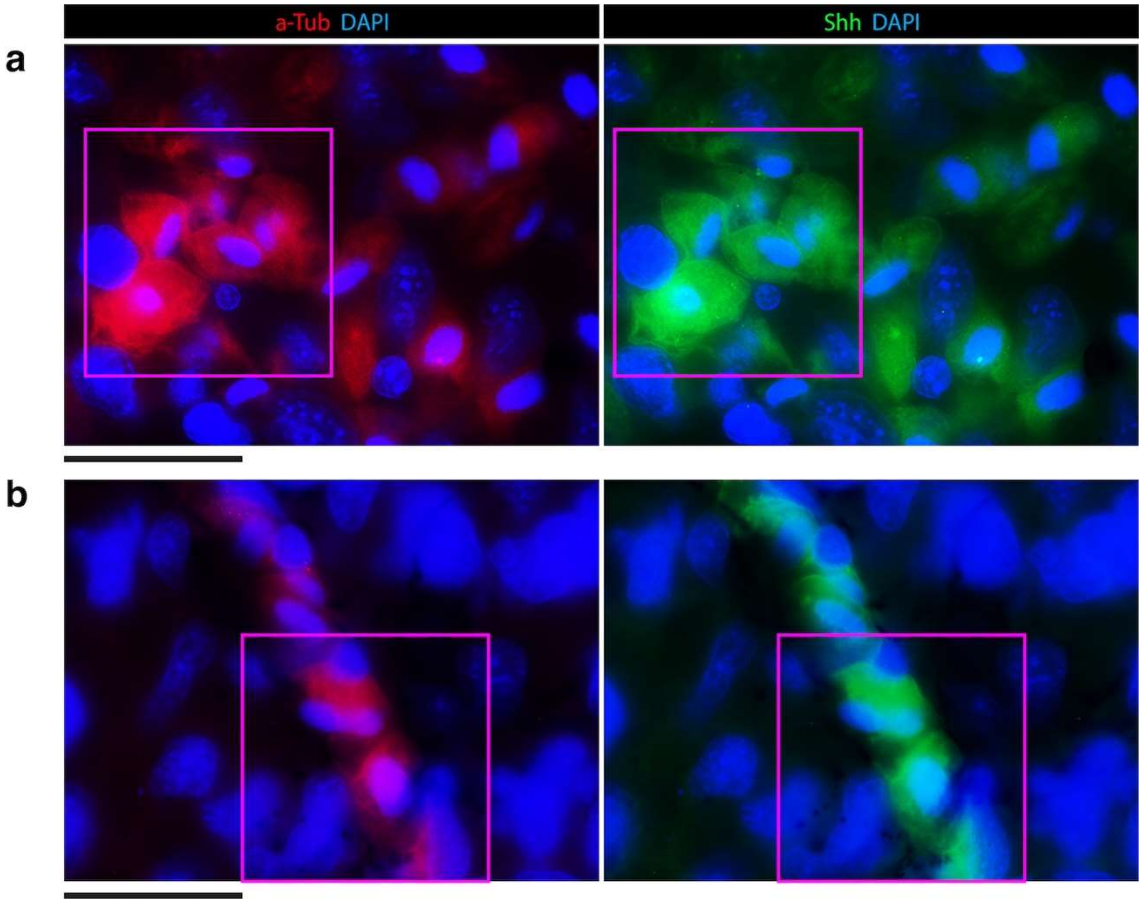

Figure S4. Expression of acetylated-tubulin and Shh protein expression in adult wild type blastema at 30 dpa. (**a**) Image enlargement of the panel shown in Figure 6b (pink inset of the blastema). Here, the unmerged image is shown. (**b**) Image enlargement of the panel shown in Figure 6e (pink inset of the blastema). Here, the unmerged image is shown. Scale bars: (**a**, **b**), 50µm.

Table S1. Conserved DNA motif/binding sites of the newt nZRS enhancer aligned with those of other vertebrates. Here, the reference mouse ZRS E0 conserved ETS site and HD (Homeodomain) are underlined [20].

Name	Strand	Start	*p*-value	E0 Conserved Site (ETS, HD, HD)
Chicken (*Gallus gallus*)	+	47	6.26e-25	CGACAGCAACATCCTGACCAATTATCCAAATTATCCAGACA
Eagle (*Aquila chrysaetos*)	+	42	6.26e-25	CGACAGCAACATCCTGACCAATTATCCAAATTATCCAGACA
Dog (*Canis lupus*)	+	47	1.13e-23	CGACAGCAACATCCTGACCAATTATCCAAGCTATCCAGACA
Newt (*Cynops orientalis*)	+	61	1.51e-23	CGACAGCAACATCCTGACTAATTACGCAAATTATCCAGACA
Newt (*Cynops pyrrhogaster*)	+	61	1.51e-23	CGACAGCAACATCCTGACTAATTACGCAAATTATCCAGACA
Axolotl (*Ambystoma mexicanum*)	+	56	5.27e-23	CGACAGCAACATCCTGACTAATTAGCCAAATTACCCAGACA
Lizard (*Anolis carolinensis*)	+	45	1.51e-22	CGACAGCAACATCCTGACCAATTATGCAAAGCATCCAGACA
Dwarf Siren (*Pseudobranchus axanthus*)	+	58	5.13e-22	CGACAGCAACATCCTGACTAATTATCCAAGTTCCCCAGACA
Mouse (*Mus musculus*)	+	45	1.74e-21	TGACAGCAcACCAATTATCCAAACCATCCAGCCA
Frog (*Kaloula pulchra*)	+	28	6.03e-21	CGGCAGAGACATCCTGACCAATTACCCAAATGACCCAGACA
Human (*Homo sapiens*)	+	40	6.46e-21	CATAAGCAACATCCTGACCAATTATCCAAACCATCCAGACA
Frog (*Xenopus tropicalis*)	+	22	9.63e-21	CAACAGCAACATCCTGGCCAATTACTCAAACGATCCAGACA
Newt (*Pleurodeles waltl*)	+	60	4.03e-18	GCGACAGCACATCCTGACTAATTACCCAAATTACCCAGACA

Table S2. Conserved DNA motif/binding sites of the newt nZRS enhancer, detected after alignment with those of other vertebrates. Here, the reference mouse limb development motif sites Pitx1 and Tbx5-Hox like are underlined [41].

Name	Strand	Start	*p*-value	Sites (Pitx1, Tbox Hox like)
Newt (*Pleurodeles waltl*)	+	135	6.46e-24	GTCTGTCGGATTAAAAGGTTAACTCCTAAAACATCAAACGA
Newt (*Cynops orientalis*)	+	136	6.46e-24	GTCTGTCGGATTAAAAGGTTAACTCCTAAAACATCAAACGA
Newt (*Cynops pyrrhogaster*)	+	136	6.46e-24	GTCTGTCGGATTAAAAGGTTAACTCCTAAAACATCAAACGA
Dog (*Canis lupus*)	+	116	4.84e-22	GTCTGTAGGATTAAGAGGTTAACTCCTATAACTTCAAACGA
Frog (*Xenopus tropicalis*)	+	75	1.17e-21	TTCTGTGGGATTAAGAGGTTAACTCCTAAAACATCAAACAA
Human (*Homo sapiens*)	+	109	2.60e-21	GTCTGTAGGATTAAGAGGTTAACTCCTATAACTTCAAACAA
Salamander (*Liua shihi*)	+	6	3.33e-21	GTCTGTCGGATTAAAAGGTTAACTCCTAAAACATCAAAAGG
Eagle (*Aquila chrysaetos*)	+	112	2.83e-19	AGTCTGTGGATTAAGAGGTTAACTCCTGGAACATCAAAAGA
Dwarf Siren (*Pseudobranchus axanthus*)	+	127	5.67e-19	ATTTGTTGAATTAAAAGGTTAACTCCTAAAAGATCAAAAGA
Mouse (*Mus musculus*)	+	114	1.14e-18	ATCTATAGGATTAAGAGGTTAGCTCCTGTAACTTCAAACAA
Chicken (*Gallus gallus*)	+	117	3.12e-18	AGTCTGTGGATTAAGAGGTTAACTCCTGGAACATCAAAGTA
Frog (*Kaloula pulchra*)	+	80	8.83e-18	TTTAGTGGGATTAAGAGGTTAATTTCTAAGACATCAAACAA
Lizard (*Anolis carolinensis*)	+	113	1.17e-17	AGTCTGTGGATTAAGAGGTTAACTTGGAGAACATCAAAAGA

Table S3. Conserved DNA motif/binding sites of the newt nZRS enhancer aligned with those of other vertebrates. Here, the reference mouse ZRS E1 conserved ETS site (known as a snake-specific deletion), HD (homeodomain), and ETV2 are underlined [20, 38]. A conserved uncharacterized E-box (CANNTG) was also found among most vertebrates.

Name	Strand	Start	*p*-value	Sites (E1 Conserved ETS1/ETV2, HD, E-box)
Salamander (*Liua shihi*)	+	75	1.80e-29	AAAAATTTGAGGTAACTTCCTTGCTTAATTAATTAGGTGGACCAGGTGGA
Newt (*Pleurodeles waltl*)	+	203	2.81e-29	AGAAATTTGAGGTAACTTCCTTGCTTAATTAATGAGGTGGGCCAGGTGGA
Chicken (*Gallus gallus*)	+	185	2.06e-28	AAAAATTTGAGGTAACTTCCTTGCTTAATTAATTAGGTAGACCAGGTGGA
Eagle (*Aquila chrysaetos*)	+	181	2.06e-28	AAAAATTTGAGGTAACTTCCTTGCTTAATTAATTAGGTAGACCAGGTGGA
Newt (*Cynops orientalis*)	+	204	3.27e-28	AGAAATTTGAGGTAACTTCCTTGCCTAATTAATGAGGTGGGCCAGGTGGA
Newt (*Cynops pyrrhogaster*)	+	204	3.27e-28	AGAAATTTGAGGTAACTTCCTTGCCTAATTAATGAGGTGGGCCAGGTGGA
Frog (*Amolops loloensis*)	+	73	9.64e-28	AAAAATGTAAGGTAACTTCCTCGCTTAATTAATTAGGTGGGCCAGGTGGA
Frog (*Kaloula pulchra*)	+	153	2.96e-27	AAAAATGTTAGGTAACTTCCTTGCTTAATTAATTAGATGGGCCAGGTGGA
Lizard (*Anolis carolinensis*)	+	184	1.24e-26	AAAATTCTGAGGTAACTTCCTTGCTTAATTAATTAGGTAGGCCAGGTGGA
Axolotl (*Ambystoma mexicanum*)	+	200	1.03e-25	AGAAATTTGAGGTAACTTCCTTGCTTAATTAATTAGCTAGGCCAGTTGGA
Salamander (Amphiuma means)	+	22	2.65e-25	AAAAAGGTAAGGTAACTTCCTCGTTTAATTAATGAGGTGGGCCAGGTGGA
Dwarf Siren (*Pseudobranchus axanthus*)	+	196	6.27e-25	AAACAATTGAGGTAACTTCCTTGCGTAATTAATTAGGTGGGCCAGGTTGA
Dog (*Canis lupus*)	+	184	8.77e-25	CAGAATTTGAGGTAACTTCCTTTCTTAATTAATTAGACTGACCAGGTGGA
Human (Homo sapiens)	+	177	2.11e-24	CAAAATTTTAGGTAACTTCCTTTCTTAATTAATTGGACTGACCAGGTGGA
Frog (*Xenopus tropicalis*)	+	176	3.01e-24	CGAAATGCAAGGTAACTTCCTAGTGTAATTAATTAGGTGGGCCAGGTGGA
Mouse (*Mus musculus*)	+	183	1.64e-22	CAAAATCTGAGGTCACTTCCTCTCTTAATTAGTTGCACTGACCAGGTGGA

Table S4. Conserved DNA motif/binding sites of the newt ZRS enhancer aligned with those of other vertebrates. This region contained a characterized mouse ETV, a regulator in the limb bud [37].

Name	Strand	Start	*p*-value	Sites<br>ETV
Chicken (*Gallus gallus*)	+	269	3.18e-30	ATAAAGCTGAGCAACATGACAGCACAATGGAGGAGGAACAAAGATTTTTT
Eagle (*Aquila chrysaetos*)	+	265	3.18e-30	ATAAAGCTGAGCAACATGACAGCACAATGGAGGAGGAACAAAGATTTTTT
Human (Homo sapiens)	+	261	1.26e-29	ATAAAGCTGAGCAACATGACAGCACAATAGAGGAGGAACAAAGATTTTTT
Newt (*Cynops orientalis*)	+	287	4.34e-29	ATAAAGCCGAGCAACATGACAGCACAATAGAGGAGAAACAAAGATTTTTT
Newt (*Cynops pyrrhogaster*)	+	287	4.34e-29	ATAAAGCCGAGCAACATGACAGCACAATAGAGGAGAAACAAAGATTTTTT
Salamander (*Liua shihi*)	+	158	1.88e-26	ATAAAGCTGAACAACATGACAGCAGAATAGAGGAGAAACAAAGATTGTTT
Lizard (*Anolis carolinensis*)	+	268	5.39e-26	ATAAAGCTGAACTATATGACAGCACAATGGAGGAGGAACAAAGATTGTTT
Newt (*Pleurodeles waltl*)	+	286	7.30e-26	ATAAAGCTGAACAACATGACAACACAATAGAGGAGAAACAACGATTTTTT
Mouse (*Mus musculus*)	+	264	5.41e-25	ATAAAGCCAAGCAACATGACAGCACAATAGAGGAGGAACTAAGATCGTTT
Dwarf Siren (*Pseudobranchus axanthus*)	+	279	7.27e-24	ATAAAGCCGAACAACATGACAGCACAATAGAGGAGAAACAACGACTTTCA
Axolotl (*Ambystoma mexicanum*)	+	283	7.84e-24	ATAAAGCTGAACAACTCGACAGCACAAAAGTGGAGAAACAAAGATTTTTT
Frog (*Amolops loloensis*)	+	158	1.87e-23	ATAAAGCTAAGTAACATGACAGCGCAATGTAGAAGAAAGCAAGATTTTTT
Dog (*Canis lupus*)	+	268	1.87e-23	ATAAAGCTGAGCACTGTGACAGCACAATGGAGGAGGAACAGAGATTGCTT
Frog (*Kaloula pulchra*)	+	238	2.28e-22	ATAAAGCTAAGTAACATGACAGCACAGTGTAGCAGAACGCAAGATTTTTT
Salamander (*Amphiuma means*)	+	105	4.33e-22	ATAAAGCCGAGTAACATGACAGGGCAAAGGAGGCCAAACCATGATTTTTT
Frog (*Xenopus tropicalis*)	+	260	1.38e-21	ATAAAGCTGGGTTACATGACAGCACCACGGCGAGGGAAGAAAGATTTTTT

Table S5. Conserved uncharacterized DNA site of the newt nZRS enhancer aligned with that of other vertebrates. This region contained a ETV2, EOMES, and Tbx4 detected in JASPAR (motif ID: MA0762.1.ETV2, UN0511.1.ETV2::EOMES, and MA0806.1.TBX4), and underlined. Conserved Hox site of the mouse ZRS [40].

Name	Strand	*p*-value	Sites<br>ETV2::EOMES Tbx4 Hox
Salamander (*Liua shihi*)	+	1.46e-30	CTATCCTGTGTCACAGTTTGAAATTGTCCTGGTTTATGTCCCTTTTGGCA
Axolotl (*Ambystoma mexicanum*)	+	1.46e-30	CTATCCTGTGTCACAGTTTGAAATTGTCCTGGTTTATGTCCCTTTTGGCA
Newt (*Pleurodeles waltl*)	+	1.46e-30	CTATCCTGTGTCACAGTTTGAAATTGTCCTGGTTTATGTCCCTTTTGGCA
Newt (*Cynops orientalis*)	+	1.46e-30	CTATCCTGTGTCACAGTTTGAAATTGTCCTGGTTTATGTCCCTTTTGGCA
Newt (*Cynops pyrrhogaster*)	+	1.46e-30	CTATCCTGTGTCACAGTTTGAAATTGTCCTGGTTTATGTCCCTTTTGGCA
Lizard (*Anolis carolinensis*)	+	1.46e-30	CTATCCTGTGTCACAGTTTGAAATTGTCCTGGTTTATGTCCCTTTTGGCA
Chicken (*Gallus gallus*)	+	1.46e-30	CTATCCTGTGTCACAGTTTGAAATTGTCCTGGTTTATGTCCCTTTTGGCA
Eagle (*Aquila chrysaetos*)	+	1.46e-30	CTATCCTGTGTCACAGTTTGAAATTGTCCTGGTTTATGTCCCTTTTGGCA
Human (Homo sapiens)	+	1.46e-30	CTATCCTGTGTCACAGTTTGAAATTGTCCTGGTTTATGTCCCTTTTGGCA
Frog (*Amolops loloensis*)	+	2.92e-30	CTATCCTGTGTCACAGTTTGAAATTGTCCTGGTTTTTGTCCCTTTTGGCA
Dog (*Canis lupus*)	+	1.03e-29	CTATCCTGTGTCACAGTGTGAAATTGTCCTGGTTTATGTCCCTTTTGGCA
Frog (*Kaloula pulchra*)	+	1.72e-29	CTATCCTGTGTCACAGTTTGAAATTGTCCTGGTTTTTGTCGCTTTTGGCA
Mouse (*Mus musculus*)	+	3.69e-29	CTATCCTGTGTCACAGTTTGAGATTGTCCTGGTTTATGTCGCTTTTGGCA
Salamander (*Amphiuma means*)	+	2.00e-28	CTTTCCTGTGTCACAGTTTGAAATTGTCCTGGTTTCTGTCGCTTTTGGCA
Frog (*Xenopus tropicalis*)	+	3.81e-27	TTATCCTGTGTCACAGTTTGAAATTGTCCTGGTTTTTCTCCCTGATGGCA
Dwarf Siren (*Pseudobranchus axanthus*)	+	6.56e-27	CTATTCTGTGTCACAGTTTGAAACTGTCCTGTGTTATGTCCCTGTTGGCA

Table S6. Conserved DNA motif/binding sites of the newt nZRS enhancer aligned with those of other vertebrates. This region contained conserved Hox sites, WMS (Werner mesomelic syndrome), and E-box previously described in the mouse ZRS [40], which are underlined. This region also contained the HAND2 mouse site [39].

Name	Strand	Start	*p*-value	Sites
				Hox WMS Hox HAND2/E-box
Newt (*Pleurodeles waltl*)	+	395	1.85e-29	AAGTTACATAAAAGTGACCCTGTACTGTATTTTATGGCCAGATGACTTTT
Newt (*Cynops orientalis*)	+	396	1.85e-29	AAGTTACATAAAAGTGACCCTGTACTGTATTTTATGGCCAGATGACTTTT
Newt (*Cynops pyrrhogaster*)	+	396	1.85e-29	AAGTTACATAAAAGTGACCCTGTACTGTATTTTATGGCCAGATGACTTTT
Chicken (*Gallus gallus*)	+	379	2.87e-29	AACTTACATAAAAGTGACCCTGTACTGTATTTTATGACCAGATGACTTTT
Eagle (*Aquila chrysaetos*)	+	375	2.87e-29	AACTTACATAAAAGTGACCCTGTACTGTATTTTATGACCAGATGACTTTT
Salamander (*Liua shihi*)	+	267	5.53e-29	AAGTTACATAAAAGTGACCCTGTACTGTATTTTATGACCAGATGACTTTT
Mouse (*Mus musculus*)	+	374	2.76e-28	AACTTACATAAAAGTGACCTTGTACTGTATTTTATGACCAGATGACTTTT
Dog (*Canis lupus*)	+	378	2.76e-28	AACTTACATAAAAGTGACCTTGTACTGTATTTTATGACCAGATGACTTTT
Human (Homo sapiens)	+	371	2.76e-28	AACTTACATAAAAGTGACCTTGTACTGTATTTTATGACCAGATGACTTTT
Lizard (*Anolis carolinensis*)	+	378	3.14e-26	AACATACATAAAAGTGACCATGTACTGTATTTTATGACCAGATGACTTTC
Frog (*Xenopus tropicalis*)	+	370	7.30e-26	AACTTACATAAAAGTGACCCTTTACTGTATTTTATAGCCAGACAACTTTT
Frog (*Amolops loloensis*)	+	270	1.71e-25	AACTTACATAAAAGTGACCCAGTACTGTATTTTATAGCCAGACGACCCTC
Salamander (Amphiuma means)	+	215	2.38e-24	AAGTTATATAAAAGTGACCATGTACTGTATTTTATAGCCAAGTGACTTTT
Axolotl (*Ambystoma mexicanum*)	+	393	6.59e-24	AGTTACAATAAAAGTGACCCTGTACTGTATTTTATGGCCAGACGACTTTT
Frog (*Kaloula pulchra*)	+	348	2.61e-23	AACTTACATAAAAGTGACCCAGTACTGTATTTTATTGCCAGACAGCCCCC
Dwarf Siren (*Pseudobranchus axanthus*)	+	387	1.31e-22	AAATTACATAAAAGTGACCCTGTACTGTATTTTATGGCCAATGACTTTTG

Table S7. Conserved DNA motif/binding sites of the newt nZRS enhancer aligned with those of other vertebrates. Here the reference mouse ZRS E3 conserved ETS site [20], Hox site [40], and ETV site [20, 37, 38].

Name	Strand	Start	*p*-value	Site
				Hox E3 ETV/ETS Conserved
Salamander (*Liua shihi*)	+	464	4.69e-30	TCCATAAATTGTTGGAAATGAGTGATTAAGGAAGTGCTGCTTAGTGTTAG
Chicken (*Gallus gallus*)	+	563	4.69e-30	TCCATAAATTGTTGGAAATGAGTGATTAAGGAAGTGCTGCTTAGTGTTAG
Eagle (*Aquila chrysaetos*)	+	558	4.69e-30	TCCATAAATTGTTGGAAATGAGTGATTAAGGAAGTGCTGCTTAGTGTTAG
Human (Homo sapiens)	+	553	4.69e-30	TCCATAAATTGTTGGAAATGAGTGATTAAGGAAGTGCTGCTTAGTGTTAG
Frog (*Amolops loloensis*)	+	458	3.05e-29	TCCATAAATTGTTGGAAATGAGTGATTAAGGAAGTGCTGCTTAGTGTTAC
Axolotl (*Ambystoma mexicanum*)	+	584	3.05e-29	TCCATAAATTGTTGGAAATGAGTGATTAAGGAAGTGCTGCTTAATGTTAG
Newt (*Cynops orientalis*)	+	589	7.50e-29	TCCATAAATAGTTGGAAATGAGCGATTAAGGAAGTGCTGCTTAGTGTTAG
Dwarf Siren (*Pseudobranchus axanthus*)	+	580	1.44e-28	TCCATAAATTGTTGGAAATGAGTGATTAAGGAAGTACTGCTTAGTGTTAG
Mouse (*Mus musculus*)	+	552	1.77e-28	TCCATAAATTGTTGGAAATGAGCGATTCAGGAAGTGCTGCTTAGTGTTAG
Newt (*Pleurodeles waltl*)	+	589	2.02e-28	TCCATAAATGGCTGGAAATGAGTGATTAAGGAAGTGCTGCTTAGTGTTAG
Newt (*Cynops pyrrhogaster*)	+	591	3.32e-28	CCCATAAATAGTTGGAAATGAGCGATTAAGGAAGTGCTGCTTAGTGTTAG
Frog (*Kaloula pulchra*)	+	531	4.72e-28	TCCATAAATTGTTGGAAATGAGTGATTAAGGAAGTGCTGCTCAGTGTTAC
Dog (*Canis lupus*)	+	559	1.05e-27	TCCATAAATTGTTGGACATGAGTGAATAAGGAAGTGCTGCTTAGTGTTAG
Frog (*Xenopus tropicalis*)	+	554	2.01e-27	TCCATAAATTGCTGGAAATGAGTGATTAAGGGAGTGCTGCTTAATGTTAG
Lizard (*Anolis carolinensis*)	+	564	9.61e-27	TCCATAAATTGTTGGGAATAAGTGATTGAGGAAGTGCTGCTTAGTGTTAG
Salamander (*Amphiuma means*)	+	405	9.56e-21	CCCATCCATAAAGTGCTGGGAATGATTAAGGAAGTGCTGCTTAATGTTAG

Table S8. Conserved DNA motif/binding sites of the newt nZRS enhancer aligned with those of other vertebrates. Here, the reference mouse ZRS E4 conserved ETS site (AGGAAATC elements) and EST1 [20, 37, 38] are underlined, noting that the human and mouse E4 sites are not shown, as blocks were not generated during MEME motif alignment. This region contained a Tbx element detected in JASPAR (motif ID: Tbx4 87 MA0806.1, TBX5 MA0807.1), shown in yellow.

Name	Strand	Start	*p*-value	Sites<br>Tbx ETV2/ETS E4 Conserved EST1
Newt (*Cynops orientalis*)	+	678	3.12e-29	GTGGGTGAGAGGAAATCATGTACTGCTGAAACAATAAGGAAGCCTCCTGC
Newt (*Cynops pyrrhogaster*)	+	677	3.12e-29	GTGGGTGAGAGGAAATCATGTACTGCTGAAACAATAAGGAAGCCTCCTGC
Chicken (*Gallus gallus*)	+	643	8.66e-29	GTGGGTGAGAGGAAATCGCGTACTGCACAAACAAAAAGGAAGACTCCTGC
Newt (*Pleurodeles waltl*)	+	678	1.88e-28	GTGGGTGAGAGGAAATCCTGTACTGCAGAAACAATAAGGAAACCTCCTGC
Eagle (*Aquila chrysaetos*)	+	638	2.19e-28	GTGGGTGAGAGGAAATCGCGTACTGCACAAACAAAAAGGAAAACTCCTGC
Dwarf Siren (*Pseudobranchus axanthus*)	+	666	3.56e-28	GTGGGTGAGAGGAAATCACGTTCTGCAGAAACAATAAGGAAACCTCCTGC
Axolotl (*Ambystoma mexicanum*)	+	680	1.73e-27	GTGGGTGAGAGGAAATCAAGTAATGCAGAAACAATAAGGAAGCCTCCTGC
Salamander (*Liua shihi*)	+	562	3.22e-26	GTTGGTGAGAGGAAATCATGTAATGCAGAAACAATAAGGAAACCTCCTGC
Frog (*Amolops loloensis*)	+	540	4.02e-24	GTGGGTGAGAGGAAATCGTGTACTTCACTAAGAAAAGGGAAGAGCCCCGC
Frog (*Xenopus tropicalis*)	+	636	2.41e-23	CTGGGTGAGAGGAAATCATGTACTGCTCCAACAATAAGGAAAGGCCCCCG
Frog (*Kaloula pulchra*)	+	613	3.31e-23	GTGGGTGAGAGGAAACCGCGTACTTCACTAAGATGAAGGAAGAGCCCCGC
Lizard (*Anolis carolinensis*)	+	646	9.81e-22	GTGGGTGAAAGGAAATCAAGCTCTGTGCAAACATAAAGGAAGATTCCTGC
Salamander (*Amphiuma means*)	+	498	1.26e-19	CTGTGAGTGAGGAAATTGTGTACTGCAGAAACAGTAAGGGCACGCGCCTG

Table S9. Generation of transgenic nZRS reporter newts at F0

DNA pg[a]/egg	Embryos injected[b]	Survival St. 38 larvae (%)	mCherry+ limb bud St. 38 larvae (%)[c]	Survival[d] St. 57–59 (%) mCherry
200	120	41 (34)	9 (22)	5 (55)
100	214	76 (35.5)	24 (31.5)	20 (83)
0	0[e]	23 (100)	-	22 (96)

[a] pg is the total mass of pnZRS-hmp-mcherry01 (Figure 1c), including the vector backbone.

[b] The injection volume was fixed at 2 nL/embryo. *I-Sce*I enzyme concentration was fixed at 2×10^{-3} U/embryo.

[c] The onset of ZRS reporter expression was detected in the St. 38 limb bud (Figure 2b), larvae were screened having expression as shown in Figures 1 and 2. A subset of larvae expressing mCherry+ cells in the limb bud (Figure 2) were reared until St. 52–59 (Figure 3), beyond metamorphosis or used for subsequent limb amputation experiments (Figures 4–6).

[d] Percentage was derived from surviving larvae in St. 38 displaying limb bud mCherry expression (c).

[e] Viability control group (n = 22/23).
